# Validation of the shortened 24-item multidimensional assessment of interoceptive awareness, version 2 (Brief MAIA-2)

**DOI:** 10.1038/s41598-023-48536-0

**Published:** 2023-12-02

**Authors:** Aleksandra M. Rogowska, Rafał Tataruch, Klaudia Klimowska

**Affiliations:** 1 Institute of Psychology, University of Opole, 45-040 Opole, Poland; 2 Faculty of Physical Education and Physiotherapy, Opole University of Technology, 758 Opole, Poland

**Keywords:** Psychology, Medical research

## Abstract

The Multidimensional Assessment of Interoceptive Awareness (MAIA) was translated into many languages and frequently used in the last decade to assess self-reported interoceptive awareness. However, many studies demonstrated weaknesses regarding unstable factor structure and poor reliability of some scales. The 24-item Brief MAIA-2 questionnaire was developed, with only three items demonstrating the highest factor loadings in each of the eight scales of the MAIA-2. The cross-sectional online study used the 37-item MAIA-2 questionnaire in a non-clinical sample of 323 people aged between 16 and 75 (*M* = 26.17, *SD* = 9.12), including 177 women (54.80%). The sample comprised 156 athletes (48.30%) and 167 non-athletes (51.70%). The Confirmatory Factor Analysis showed adequate fit indices for a multidimensional model of the Brief MAIA-2, with the original eight scales: Noticing (awareness of subtle bodily sensations, such as the heartbeat, digestive sensations, or the breath), Not Distracting (ability to maintain attention to bodily sensations without being easily distracted by external stimuli), Not Worrying (tendency to not be overly concerned or anxious about bodily sensations or changes in the body), Attention Regulation (ability to regulate attention to bodily sensations and to shift attention between internal and external stimuli), Emotional Awareness (awareness and understanding of how emotions are associated with bodily sensations), Self Regulation (ability to regulate emotional responses and manage distress through an awareness of bodily sensations), Body Listening (tendency to listen to the body for insight and understanding), and Trusting (trust in bodily sensations as a source of information about one's feelings and needs). The hierarchical bi-factor (S·I – 1) model showed even better-fit indices. Therefore, the general factor of interoception was considered in further statistical tests. Confirmatory composite analysis showed high reliability and discriminant and convergent validity for most Brief MAIA-2 scales, except Noticing. Measurement invariance was confirmed across genders (Women, Men) and sports participation (Athletes, Non-athletes). However, group differences were also found for mean scores in particular scales of the Brief MAIA-2. Men scored significantly lower than women in Not Distracting but higher in Not Worrying, Attention Regulation, Self Regulation, Trusting, and the total score of interoceptive awareness. Gender discrepancies may be influenced by linguistic socialization, which tends to categorize shifts in internal states as either physiological or emotional. Athletes scored significantly lower than Non-athletes on the Not Distracting scale, but they showed higher scores in Noticing, Attention Regulation, Emotion Awareness, Self-Regulation, Body Listening, Trusting, and the global score, suggesting that physical training can improve most areas of interoception. Therefore, physical exercises and mindfulness training may be recommended to improve interoception, especially in women and people suffering from somatic and mental problems. The Brief MAIA-2 is a reliable and valid tool to measure multidimensional interoceptive sensibility in a non-clinical population. To improve well-being and athletic performance, Brief MAIA-2 can be used to assess the body's current perception of interoception and to detect its weak areas requiring improvement. However, the study has some limitations, such as a cross-sectional online self-report survey in a conventional non-clinical sample from Poland. Future cross-cultural studies should include representative samples for non-clinical and clinical populations from different countries and geographic regions to compare the Brief MAIA-2 with more objective psychophysiological methods of measuring interoception to reduce the limitations of these studies.

## Introduction

### Interoception characterization

Interoception refers to the processing of internal physiological states of the body by the nervous system^1^. This sense allows individuals to monitor and interpret their internal bodily states, contributing to their self-awareness and understanding of emotional and physical experiences. Interoception plays a crucial role in regulating various bodily functions and emotions. For example, recognizing the signals of hunger or thirst allows people and animals to react appropriately. Similarly, interoception is linked to emotional awareness, as certain bodily sensations can be associated with specific emotions. Therefore, people with a well-developed sense of interoception are often more in touch with their emotions and better equipped to manage stress, anxiety, and other emotional states.

Garfinkel et al.^2^ proposed a three-dimensional model of interoception, which includes interoceptive accuracy (the ability to detect and track internal sensations accurately), interoceptive sensibility (self-reported tendency to focus on internal sensations and the detection ability), and interoceptive awareness (the correspondence between objective interoceptive accuracy and self-report interoceptive sensibility). It is the degree to which interoceptive accuracy (assessed in various task performances) can be predicted from subjective self-report confidence in one's behavior performance. The sensing, interpretation, and integration of interoceptive signals originating from within the body may be processed at conscious or unconscious levels of metacognition^3^. Dimensions of interoception include lower and higher-order processing. Lower levels of interoceptive processing cover the visceral, neural, and preconscious impact of afferent signals in the central nervous system, while higher-order levels refer to interpretational dimensions of interoceptive accuracy, self-report of interoceptive awareness (sensitivity), and beliefs (both conscious and unconscious) about interoceptive aptitude, experiences, and sensations, and also interoceptive insight (metacognitive confidence of correspondence of self-report and behavioral measures), attention to and attribution of interoceptive sensations.

Regarding gender differences in interoception, women usually demonstrate poorer interoceptive accuracy but better emotional processing and recognition than men^4–7^. Interoceptive processes regulate physical exertion in physically active people, enhancing their physical and mental health and overall well-being^8^. Participating in the exercise intervention program can improve interoceptive awareness in healthy adults^9,10^, children^11^, and war veterans^12^. Research suggests that participating in sports also has a beneficial effect on interoceptive awareness^13,14^. The study^15^ performed during the COVID-19 pandemic showed that athletes who exhibited high levels of body trust demonstrated an ability to regulate their perceived stress levels by increasing positive emotions and decreasing negative stress reactions. The findings suggest that engaging in mindful activities related to the body can be beneficial for athletes in reducing perceived stress and coping with unprecedented situations during the pandemic.

### Interoception measurement

Interoception can be measured using various methods designed to assess an individual's ability to perceive and interpret their internal bodily sensations, such as Heartbeat Perception Task (HPT)^16^, Heart Rate Variability (HRV)^17^, Biofeedback Devices^18^, and Behavioral Tasks (tasks involved in assessing an individual's ability to perceive changes in bodily sensations in response to physical exertion, heat, cold, or other stimuli)^19^. Several self-report questionnaires have also been developed to assess interoceptive awareness, including Body Awareness Questionnaire (BAQ)^20^, Body Perception Questionnaire (BPQ)^21^, Interoceptive Confusion Questionnaire (ICQ)^22^, and Interoceptive Accuracy Scales (IAS)^23^. These questionnaires inquire about various sensations and perceptions related to bodily processes, including subjective awareness of sensations like heartbeat, breathing, and digestive processes, providing a comprehensive view of an individual's interoceptive abilities.

One of the most commonly used self-report instruments in the last decade is the Multidimensional Assessment of Interoceptive Awareness (MAIA)^24–26^. The MAIA measures interoceptive sensibility, understood as the self-perceived tendency to focus on interoceptive stimuli^1^. This questionnaire aims to provide a comprehensive understanding of interoceptive awareness by capturing various facets of this complex phenomenon, including noticing (an individual's ability to notice and pay attention to their bodily sensations), not-distracting (the tendency to avoid distraction from internal sensations or to avoid using external distractions to suppress those sensations), not-worrying (the tendency not overly to worry or be anxious about internal bodily sensations), attention regulation (the level of attention and responsiveness to bodily sensations), emotional awareness (the capacity to be aware of how bodily sensations relate to emotional states), self-regulation (ability to regulate physiological processes based on their interoceptive cues), body listening (the level of attention and responsiveness to bodily sensations), and trusting (the extent to which an individual trusts their internal bodily sensations). The MAIA has been used in research to investigate interoceptive sensitivity in various populations, including clinical groups^27–34^ and healthy individuals^24,35–52^.

MAIA was used in many previous studies related to contemplative main-body interventions^30,31,36^, mindfulness^39,46,49–51,53^, physical activity^8–11,13,14,24,37^, emotional susceptibility^38^, emotion recognition^54^, emotion regulation^49^, alexithymia^29,39^, anxiety^27,29,41,46,49,50^, depression^27,28,30,31^, pain^28,32,49^, somatization^27^, eating disorders symptoms^27,29,37^, personality^39,41,55,56^, self-esteem^51^, and well-being^53^. MAIA was validated and adapted to many languages, including Arabic^40^, Chinese^45,50^, French^39^, German^33,36^, Hungarian^41^, Italian^38^, Japanese^43,49^, Korean^44^, Lithuanian^35^ Malay^51^, Norwegian^42^, Polish^37^, Portuguese^46^, Spanish^47,52^, and Turkish^48^.

However, some previous validation studies using CFA showed unacceptable fit indices for multidimensional 8-factor structure^29,32,35,39^, and poor reliability, usually for scales Noticing^31,33,35,37,38,41,50,52,53^, Not Distracting^29,31–33,35–38,41,45,49,52^, Not Worrying^29,31–33,35–38,43,45,50,52,53^, and Trusting^48^. In addition, Valenzuela-Moguillansky et al.^52^ removed two items (4 and 8), while Cali et al.^38^ excluded five items (4, 7, 10, 12, and 19) from MAIA to improve the 8-factor structure. A 7-factor solution of EFA was found in several studies^46,47,50^. In the Portuguese sample, Machorrinho et al.^46^ showed a 7-factor solution in EFA (without Body Listening scale). Similarly, Montoya-Hurtado^47^ showed a 7-factor structure by removing the Not Worrying scale and item 6 of the MAIA. Teng et al.^50^ removed six items, and the Noticing scale and good fit indices were achieved by modification (residual correlations between some items). Furthermore, a 6-factor solution was found using EFA in several studies^35,39,41,43,44,48,49^, with an exclusion of some scales or particular items (e.g., Not Distracting and Not Worrying scales were excluded in French and Hungarian studies, Noticing and Self Regulation scales were deleted with five items in Turkish sample, while Not Worrying and Self Regulation scales were removed together with seven items from Japanese validation research). Finally, recent Malay translation and validation showed a 3-factor structure by excluding 13 items from MAIA questionnaire^51^.

Although most studies examined only multidimensional factor structure (all scales of the MAIA are intercorrelated), some researchers examined higher-order general factors using CFA^39,41,42^, including hierarchical second-order (selected items are related to specific scales and all eight scales of MAIA are related to the general factor of interoceptive awareness on a higher level) and bi-factor structures (all items of MAIA are related to the general factor, and selected items to eight scales, independently). Fiskum et al.^42^ confirmed that both second-order and bi-factor models of MAIA-2 have appropriate structure and good fit indices, so the MAIA-2 total score can be considered in further studies as an indicator of a general factor of interoception. However, resolutions of such an approach did not always cover all items and scales of MAIA^39,41^, and were far from theoretical assumptions^24–26^. For example, Ferentzi et al.^41^ excluded two scales (Not Distracting and Not Worrying) from the general factor because of the previous weak correlations with the other MAIA scales. Therefore, only six factors were tested for the general factor of self-report interoception in the bi-factor model. Furthermore, Da Costa Silvia et al.^39^ found that the second-order solution fit the data well, but only for 6-factor instead of the original 8-factor structure (excluding two scales: Not distracting and Not worrying).

Gender invariance was confirmed in the Arabic adaptation of the MAIA-2^40^ and Malay validation of the MAIA^51^. However, the Student's *t*-test showed statistically significant gender differences in two scales: Not Worrying and Attention Regulation. Men scored significantly higher than women in these dimensions^40^. Fiskum et al.^42^ performed MANCOVA, showing that women scored significantly higher than men in the total MAIA-2 and Trusting scales, but they presented lower scores in Not Worrying than men.

### The present study

**Figure 1:**
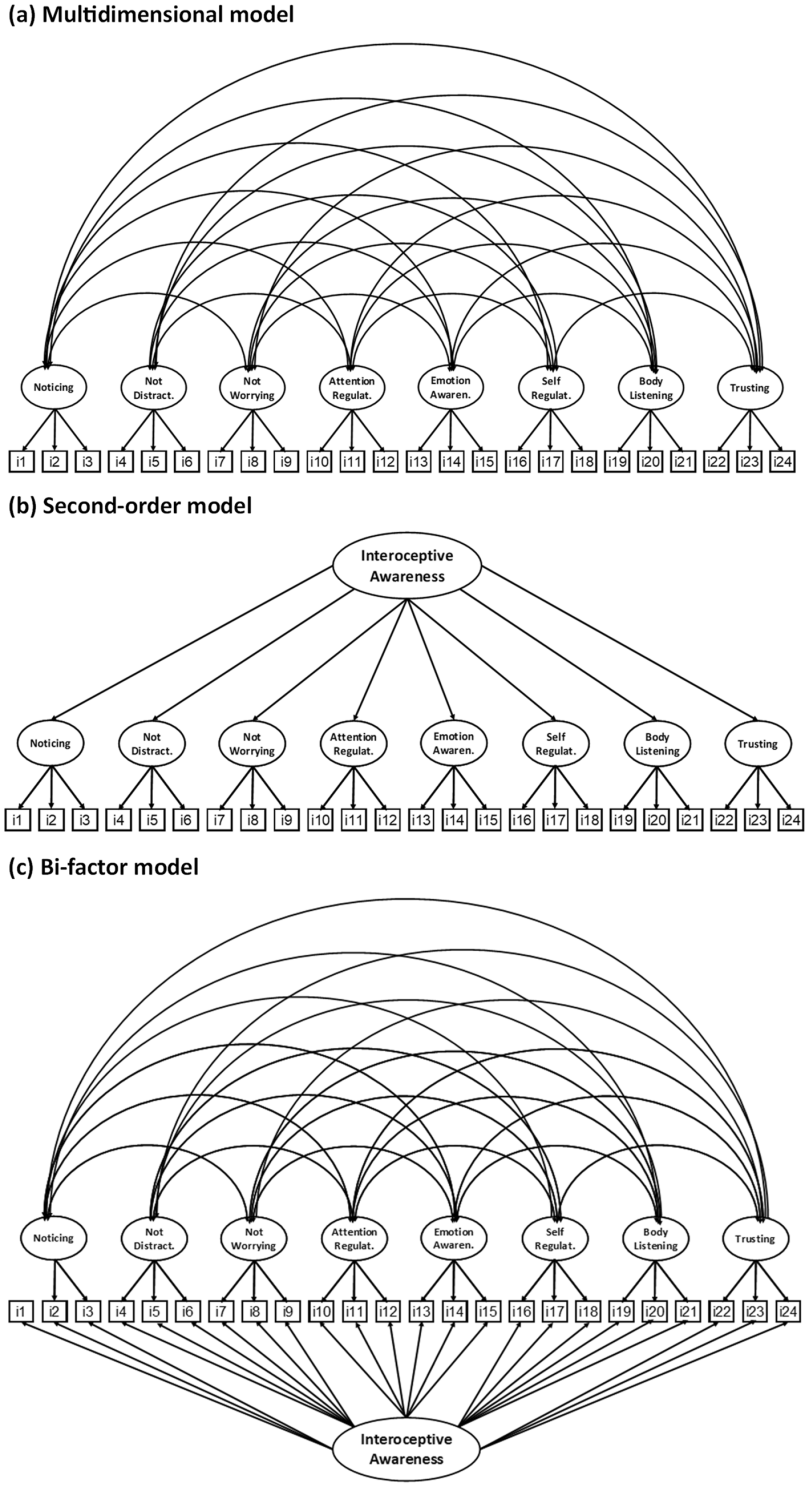
Three hypothetical factor models of the 24-item Brief MAIA-2: (**a**) Multidimensional model, (**b**) Second-order model, and (**c**) Bi-factor model.

Although MAIA was previously translated into Polish and used in one study^37^, the factor structure of the questionnaire was not validated. In addition, three scales were presented with poor internal consistency (Cronbach's α < 0.70). However, the reliability can be questionable, considering that the study sample was small (*N* = 75). Therefore, we translated, adapted, and validated the recently modified version of the MAIA-2 questionnaire. Previous studies showed various validation issues, including unstable factor structure in EFA (from three to ten factors), numerous modification indices in CFA that can improve model fit, and poor reliability (Cronbach's α below threshold of 0.70). We assumed that the multidimensional MAIA-2 might contain items not strongly associated with a particular dimension or total construct of interoceptive awareness and that some items may share a common variance across scales. To prevent these problems, we developed a short version of the MAIA-2 that contains only the three items with the highest factor loadings on each of the eight scales, originally designed by Mehling et al.^24^. A minimum of three items are required to calculate credible assessment of reliability (e.g., Cronbach's α or McDonald's ω). Using CFA, we will examine three various factor structures: multidimensional (Fig. 1a), second-order (Fig. 1b), and bi-factor (Fig. 1c) models of the 24-item Brief MAIA-2. Also, composite CFA will examine reliability, convergent, and discriminant validity in the Brief MAIA-2. Furthermore, measurement invariance will be tested to check whether gender and physical activity moderate the factor structure found in CFA. Finally, gender and physical activity differences will be examined in mean scores of the Brief MAIA-2 scales. We hypothesize that shortening the MAIA-2 improves all fit indices of the 8-factor structure. We assume, based on previous studies^4–11,13,14,24,40,42^, that there are intergroup differences in mean scores of Brief MAIA-2 scales, but these differences do not contribute to the factor structure of interoceptive awareness ^40,51^.

## Methods

### Study design and procedure

The cross-sectional online study recruited participants two times. The first research was performed between 3 August 2020 and 30 November 2020 among university students of Physical Education and elite athletes in speed skating. An invitation to the online survey (prepared in Google Forms) was disseminated to both groups by e-mail. The second recruitment included a control group of non-athletes. The research was conducted between July 2021 and March 2022 via the Facebook social network on various groups: *Student Surveys, Psychology UO 2017/2022, Clinical Psychology UO, Research, surveys, interviews, Students and graduates of the Opole University of Technology, Men also feel, Positive psychology, Integration group, Health without drugs, and Motorcycle expeditions*. The link to the online survey was also shared privately by a person on Facebook after random selection. Before starting the study, the questionnaire contained information about the purpose of the study, the anonymity and confidentiality of the results, and the possibility of resignation at any time.

### Measure

The Multidimensional Assessment of Interoceptive Awareness, version 2 (MAIA-2), is a self-report questionnaire designed to measure various dimensions of an individual's interoceptive awareness. Developed by Mehling et al.^24,25^, it assesses different aspects of how people perceive and relate to their internal bodily sensations. The MAIA-2 questionnaire consists of 37 items and eight subscales, each focusing on a specific dimension of interoceptive awareness: Noticing (items 1–4), Not Distracting (items 5–10), Not Worrying (items 11–15), Attention Regulation (items 16–22), Emotional Awareness (items 23–27), Self Regulation (items 28–31), Body Listening (items 32–34), and Trusting (items 35–37). The MAIA questionnaire is presented as a series of statements or items related to these dimensions, and participants are asked to rate their level of agreement with each statement using a 6-point Likert scale (from 0 = *Never* to 5 = *Always*). A higher score (0–185) indicates greater interoceptive sensibility. Cronbach's alpha ranged between 0.64 and 0.83 in the previous study^24^. We independently used the forward–backward translation process from English to Polish (and vice versa) by two experts (one was a psychology professor from a Polish university, and the d second was an English native speaker). Each item was discussed and corrected if necessary. A pilot study was performed on 12 psychology students who participated in a Master's seminary. We modified some items according to the suggestions and commentaries of the Pilot Sample. The final 37-item version of MAIA-2 was conducted in the online survey (See Supplementary Materials for the Polish version of the MAIA-2 and raw data).

### Participants

A total sample of 323 people participated in the study. The age of individuals ranged between 16 and 75 (*M* = 26.17, *SD* = 9.12). The sample includes 143 men (44.27%) and 177 women (54.80%), while three people preferred not to indicate their gender (0.93%). Among athletes (*n* = 156, 48.30%), 102 (32.58%) were Physical Education students, and 54 (16.72%) were elite athletes in speed skating. The sample of non-athletes (*n* = 167, 51.70%) consisted of 57 (17.65%) students (high school, college, or university), 100 (30.96%) working people, 7 (2.17%) unemployed and three retirees (0.93%).

### Statistical analysis

Parametric properties of all scales and items of the MAIA-2 were examined using a range of scores, mean score (*M*), standard deviation (*SD*), median (*Mdn*.), skewness, and kurtosis. Since all scales ranged in skewness and kurtosis ± 1, and the sample size was large (*N* > 200), we assumed that variables present appropriate properties for further parametric analyses^57^. Initially, we conducted the correlation matrix between all items of the MAIA-2, the Kaiser–Meyer–Olkin (KMO), and Bartlett's test of sphericity to explore whether the data are appropriate for factor analysis. We conducted the confirmatory factor analysis (CFA) with the maximum likelihood (ML) estimation method to examine the structure of MAIA-2 in the total sample (*N* = 323). Next, we selected three items per each of the eight scales of the MAIA-2, which were shown with the highest values of factor loadings, to prepare the 24-item Brief MAIA-2 version of the questionnaire. Then, we conducted CFA again to compare the fit indices of three various models: (1) multidimensional model, (2) hierarchical second-order model, and (3) bi-factor model. However, due to Eid et al.'s^58^ suggestions and argumentation, we performed a bi-factor (S·I – 1) model, which is better defined for this study's single-level sampling design in this study. In the bi-factor (S·I – 1) model, the first item of the Brief MAIA-2 is not loaded to the Noticing scale but to the general factor of Interoceptive awareness.

Construct validity was evaluated also using several goodness-of-fit criteria, including ML χ^2^, *df* and *p*-value (the ratio χ^2^/*df* < 2 is considered very good fit, between 2 and 3 – good, and acceptable < 5), standardized root mean squared residual (SRMR ≤ 0.08 is acceptable), root mean square error of approximation (RMSEA; acceptable fit if ≤ 0.08, adequate fit if < 0.06, and good if 0.04), comparative fit index (CFI is acceptable if ≥ 0.90, and good if > 0.95)^59^. The measurement invariance (MI) was examined using multigroup CFA (MGCFA) to check whether Brief MAIA-2 scores in the latent variable and particular items varied across genders and sports participation. We conducted hierarchical tests for the invariance of measurement parameters, assuming more equality restrictions in each consecutive model in the following sequence: configural, metric, scalar, and strict MI models. Configural invariance verified that the same CFA structure is valid in each gender or sport participating group. Metric invariance assumes that factor loadings are equal across groups, examining whether participants across groups attribute the same meaning to the latent construct under study. The scalar invariance test of the null hypothesis examines whether the loadings and intercepts are constrained to be equal. Scalar invariance implies that the meaning of the construct (factor loadings) and the underlying items (intercepts) levels are equal in all groups, meaning scores on the latent variable are comparable across groups. Strict invariance constrained factor loadings, item intercept, and residual variances to be equal across groups. If strict invariance is confirmed, the latent construct is measured identically across groups. Chen^60^ suggests a change of ≤ –0.005 in CFI, supplemented by a change of ≥ 0.010 in RMSEA, as an indicator of non-invariance when the compared sample sizes are unequal. For testing intercept or residual invariance, a change of ≥ –0.005 in CFI, supplemented by a change of ≥ 0.010 in RMSEA, would indicate non-invariance.

Finally, the correlation analysis was performed to examine associations between particular scales of the Brief MAIA-2. The reliability of scales was assessed using McDonald's *ω* and Cronbach's *α*, which can be classified as poor if its values are below 0.60, as questionable if between 0.60 and 0.70, as acceptable if between 0.70 and 0.80, as good if between 0.80 and 0.90, and above 0.90 as excellent^57^. The confirmatory composite analysis (CCA) was also conducted to evaluate the CFA structure's convergent and discriminant validity, which considers sampling errors for the psychometric quality measures. The CCA is assessed using several indicators, including correlations between latent variables (*r* ≤ 0.80 means good discriminant validity), construct reliability (CR ≥ 0.70 denotes good reliability), average variance extracted (AVE ≥ 0.50 represent an acceptable level of convergent validity), and heteotrait-monotrait (HTMT ≤ 0.85 indicates good discriminant validity)^61,62^. Criterion validation was examined using independent samples Student's *t*-test for gender and sports participation differences. Convergent and discriminant validity was examined using independent samples Student's *t*-test for gender and sports participation. All statistical tests were performed using IBM SPSS ver. 26 and AMOS ver. 26. The CCA analysis was performed using the "Master Validity Tool" for AMOS ver. 26^63^.

### Informed consent

Informed consent was obtained from all subjects involved in the study.

### Institutional review board statement

The study was conducted according to the guidelines of the Declaration of Helsinki and approved by the Scientific Research Ethics Committee of the University of Opole (decision No. 6/2020, 13 July 2020).

## Results

### Confirmatory factor analysis for MAIA-2

The Kaiser–Meyer–Olkin (KMO) test was 0.92, while Bartlett's test of sphericity yielded χ^2^(666) = 7086.18 (*p* < 0.001), which suggests that factor analysis would be useful with the data^57^. The CFA with ML estimation method was performed for the multidimensional model of MAIA-2, including all 37 items. The multidimensional model showed acceptable fit indices for χ^2^/*df* = 2.64, *p* < 0.001, and RMSEA = 0.071 (90% CI = 0.067, 0.076), but unacceptable for SRMR = 0.102 and CFI = 0.854. Factor loadings are presented in Table 1. We selected three items with the highest standardized estimates and the lowest standard errors for each scale of the MAIA-2 for a further Brief version of this questionnaire.

**Table 1:** Parameter estimates for the Multidimensional Assessment of Interoceptive Awareness, version 2 (MAIA-2).

Factor	Indicator	Estimate	*SE*	Std. Est	*Z*	*p*	95% CI	
							LL	UL
Noticing	MAIA-2_01	0.94	0.09	0.60	10.60	< 0.001	0.76	1.11
	MAIA-2_02^#^	0.88	0.08	0.63	10.94	< 0.001	0.72	1.04
	MAIA-2_03^#^	1.01	0.09	0.65	11.31	< 0.001	0.84	1.19
	MAIA-2_04^#^	0.96	0.08	0.66	12.04	< 0.001	0.80	1.11
Not Distracting	MAIA-2_05	0.74	0.08	0.54	9.72	< 0.001	0.59	0.89
	MAIA-2_06^#^	0.83	0.07	0.61	11.24	< 0.001	0.68	0.97
	MAIA-2_07	0.63	0.07	0.51	8.99	< 0.001	0.49	0.76
	MAIA-2_08	0.89	0.08	0.61	11.18	< 0.001	0.74	1.05
	MAIA-2_09^#^	1.05	0.06	0.82	16.71	< 0.001	0.93	1.17
	MAIA-2_10^#^	1.06	0.07	0.79	15.96	< 0.001	0.93	1.19
Not Worrying	MAIA-2_11^#^	1.20	0.07	0.84	16.57	< 0.001	1.06	1.34
	MAIA-2_12^#^	1.13	0.07	0.82	16.05	< 0.001	0.99	1.27
	MAIA-2_13	0.13	0.08	0.09	1.53	0.127	-0.04	0.29
	MAIA-2_14	0.03	0.08	0.02	0.37	0.715	-0.13	0.19
	MAIA-2_15^#^	0.84	0.07	0.65	12.08	< 0.001	0.71	0.98
Attention Regulation	MAIA-2_16	1.04	0.07	0.72	14.49	< 0.001	0.90	1.19
	MAIA-2_17^#^	1.10	0.06	0.81	17.19	< 0.001	0.97	1.23
	MAIA-2_18	0.99	0.07	0.73	14.76	< 0.001	0.86	1.12
	MAIA-2_19^#^	1.09	0.06	0.82	17.62	< 0.001	0.97	1.21
	MAIA-2_20^#^	1.09	0.06	0.82	17.75	< 0.001	0.97	1.21
	MAIA-2_21	1.00	0.06	0.78	16.34	< 0.001	0.88	1.12
	MAIA-2_22	1.10	0.07	0.76	15.88	< 0.001	0.96	1.23
Emotional Awareness	MAIA-2_23	0.98	0.08	0.66	12.56	< 0.001	0.83	1.13
	MAIA-2_24	0.98	0.08	0.68	13.08	< 0.001	0.83	1.12
	MAIA-2_25^#^	1.17	0.07	0.82	17.34	< 0.001	1.04	1.31
	MAIA-2_26^#^	1.12	0.07	0.79	16.25	< 0.001	0.99	1.26
	MAIA-2_27^#^	1.15	0.07	0.79	16.35	< 0.001	1.02	1.29
Self Regulation	MAIA-2_28	1.13	0.07	0.78	15.99	< 0.001	0.99	1.27
	MAIA-2_29^#^	1.12	0.07	0.80	16.71	< 0.001	0.99	1.25
	MAIA-2_30^#^	1.18	0.07	0.79	16.47	< 0.001	1.04	1.32
	MAIA-2_31^#^	1.18	0.07	0.83	17.81	< 0.001	1.05	1.31
Body Listening	MAIA-2_32^#^	0.95	0.07	0.71	13.39	< 0.001	0.81	1.09
	MAIA-2_33^#^	1.08	0.08	0.71	13.58	< 0.001	0.93	1.24
	MAIA-2_34^#^	1.16	0.07	0.83	16.63	< 0.001	1.02	1.30
Trusting	MAIA-2_35^#^	1.40	0.07	0.92	20.85	< 0.001	1.27	1.53
	MAIA-2_36^#^	1.35	0.07	0.89	20.09	< 0.001	1.22	1.49
	MAIA-2_37^#^	1.13	0.07	0.77	16.03	< 0.001	0.99	1.27

^#^Items selected for the Brief MAIA-2 are marked by hash.

### Construct Validity for the Brief MAIA-2

The CFA was performed for the 24-item Brief MAIA-2 version of the questionnaire. Factor loadings for the multidimensional model of the Brief MAIA-2 are presented in Table 2. We conducted CFA again for comparison of three models of the Brief MAIA-2: (1) multidimensional model, (2) hierarchical second-order model, and (3) bi-factor (S·I – 1) model (Table 3). The hierarchical second-order model demonstrates inappropriate fit indices, while the multidimensional and bi-factor (S·I – 1) models show good properties. However, the best indices are presented in the bi-factor (S·I – 1) model of the Brief MAIA-2.

**Table 2:** Fit indices for the Brief MAIA-2 models.

Models	χ^2^/(*df*)	SRMR	RMSEA (90% CI)	CFI
Multidimensional	2.406	0.066	0.066 (0.059, 0.073)	0.926
Second-order	8.080	0.126	0.148 (0.142. 0.154)	0.576
Bi-factor (S·I – 1)	2.128	0.048	0.059 (0.051, 0.067)	0.946

**Table 3:** Parameter estimates for the Brief Multidimensional Assessment of Interoceptive Awareness, version 2 (Brief MAIA-2).

Factor	Indicator	Estimate	SE	Std. Est	*Z*	*p*	95% CI	
							LL	UL
Noticing	MAIA-2_02	0.81	0.09	0.57	9.42	< 0.001	0.64	0.97
	MAIA-2_03	1.05	0.09	0.67	11.41	< 0.001	0.87	1.23
	MAIA-2_04	0.97	0.08	0.68	11.64	< 0.001	0.81	1.13
Not distracting	MAIA-2_06	0.78	0.08	0.57	10.25	< 0.001	0.63	0.93
	MAIA-2_09	1.09	0.07	0.85	16.50	< 0.001	0.96	1.22
	MAIA-2_10	1.08	0.07	0.81	15.56	< 0.001	0.94	1.22
Not worrying	MAIA-2_11	1.19	0.07	0.83	16.28	< 0.001	1.05	1.33
	MAIA-2_12	1.14	0.07	0.82	16.09	< 0.001	1.00	1.28
	MAIA-2_15	0.84	0.07	0.65	12.00	< 0.001	0.70	0.97
Attention regulation	MAIA-2_17	1.07	0.07	0.78	15.99	< 0.001	0.94	1.20
	MAIA-2_19	1.08	0.06	0.81	16.86	< 0.001	0.96	1.21
	MAIA-2_20	1.13	0.06	0.85	18.03	< 0.001	1.01	1.25
Emotional awareness	MAIA-2_25	1.14	0.07	0.80	16.30	< 0.001	1.00	1.27
	MAIA-2_26	1.20	0.07	0.85	17.90	< 0.001	1.07	1.33
	MAIA-2_27	1.18	0.07	0.81	16.81	< 0.001	1.04	1.32
Self regulation	MAIA-2_29	1.08	0.07	0.77	15.77	< 0.001	0.95	1.22
	MAIA-2_30	1.21	0.07	0.81	16.96	< 0.001	1.07	1.35
	MAIA-2_31	1.20	0.07	0.85	18.06	< 0.001	1.07	1.33
Body listening	MAIA-2_32	0.93	0.07	0.69	13.00	< 0.001	0.79	1.07
	MAIA-2_33	1.08	0.08	0.71	13.63	< 0.001	0.93	1.24
	MAIA-2_34	1.18	0.07	0.85	17.04	< 0.001	1.04	1.32
Trusting	MAIA-2_35	1.40	0.07	0.92	20.83	< 0.001	1.27	1.53
	MAIA-2_36	1.35	0.07	0.89	20.05	< 0.001	1.22	1.48
	MAIA-2_37	1.13	0.07	0.78	16.04	< 0.001	0.99	1.27

### Confirmatory composite analysis and reliability of the Brief MAIA-2

Initially, we examined the parametric properties of newly developed scales of the Bries MAIA-2 (Table 4). Reliability was appropriate for all scales of the Brief MAIA-2, excluding Noticing (both Cronbach's α and McDonald's ω were below the threshold of 0.70 but close to it, indicating good convergent validity of scales (Table 4). Considering the correlation matrix (Table 4), all scales were intercorrelated at r < 0.80, showing good discriminant validity. Although almost all scales were interrelated at weak or moderate Pearson's *r* values (*p* < 0.001), the Not Worrying scale was not correlated with Not Distracting, Attention Regulation, Self Regulation, Trusting, and Total score of interoceptive awareness. The general factor of interoception was the most strongly related to Self Regulation, Trusting, Attention Regulation, and Body Listening.

**Table 4:** Parametric properties for scales of the Brief MAIA-2 (*N* = 323).

Scale	Range	*Mdn*	*M*	*SD*	Skew	Kurt	α	ω
1. Noticing	0–15	10	9.66	3.44	− 0.51	− 0.32	0.67	0.68
2. Not Distracting	0–15	7	7.26	3.31	0.30	− 0.31	0.78	0.79
3. Not worrying	0–15	7	7.01	3.51	0.17	− 0.66	0.81	0.82
4. Attention regulation	0–15	9	8.05	3.55	− 0.31	− 0.61	0.86	0.86
5. Emotion awareness	0–15	11	10.03	3.80	− 0.58	− 0.32	0.86	0.86
6. Self Regulation	0–15	8	7.21	3.78	− 0.26	− 0.86	0.85	0.85
7. Body Listening	0–15	9	8.18	3.59	− 0.33	− 0.55	0.79	0.79
8. Trusting	0–15	9	8.87	4.09	− 0.37	− 0.74	0.89	0.90
9. Total interoception	24–103	67	66.27	15.59	− 0.19	− 0.49	0.84	0.88

Scale	Pearson's correlations							
	1	2	3	4	5	6	7	8
2. Not distracting	− 0.32***							
3. Not worrying	− 0.32***	0.10						
4. Attention regulation	0.45***	− 0.38***	− 0.04					
5. Emotion awareness	0.60***	− 0.28***	− 0.30***	0.43***				
6. Self regulation	0.37***	− 0.38***	− 0.02	0.64***	0.43***			
7. Body listening	0.46***	− 0.26***	− 0.30***	0.48***	0.56***	0.58***		
8. Trusting	0.38***	− 0.30***	− 0.02	0.60***	0.39***	0.67***	0.48***	
9. Total interoception	0.63***	− 0.22***	0.02	0.76***	0.68***	0.80***	0.72***	0.78***

α = Cronbach’s α, ω = McDonald’s ω. ***p < 0.001.

Convergent and discriminant validity was also assessed using confirmatory composite analysis (CCA), as shown in Table 5. All scales (except Noticing) demonstrated good reliability (CR ≥ 0.70) and convergent validity (AVE ≥ 0.50). Discriminant validity was found in all scales of the Brief MAIA-2 (HTMT ≤ 0.85).

**Table 5:** Convergent and discriminant validity for Brief MAIA − 2.

Brief MAIA-2		CR	AVE	HTMT							
				1	2	3	4	5	6	7	8
1	Noticing	0.67	0.41	1.00							
2	Not Distracting	0.86	0.56	0.44	1.00						
3	Not Worrying	0.79	0.61	0.39	0.08	1.00					
4	Attention Regulation	0.81	0.67	0.57	0.47	0.05	1.00				
5	Emotion Awareness	0.86	0.67	0.78	0.34	0.36	0.50	1.00			
6	Self Regulation	0.90	0.66	0.39	0.46	0.02	0.75	0.49	1.00		
7	Body Listening	0.85	0.56	0.61	0.33	0.37	0.58	0.69	0.71	1.00	
8	Trusting	0.79	0.75	0.37	0.36	0.02	0.68	0.44	0.77	0.56	1.00

### Group differences in mean scores of the Brief MAIA-2

The independent samples Student's *t*-test was performed to examine gender differences in the Brief MAIA-2 scale (Table 6) as a criterion validation. Women scored significantly higher than men in Not Distracting (small effect size, *p* < 0.01), while men outperform women in Not Worrying (small effect size, *p* < 0.01), Attention Regulation (medium effect size, *p* < 0.001), Self Regulation (medium effect size,* p* < 0.001), Trusting (large effect size, *p* < 0.001), and the total score of interoceptive awareness (small effect size,* p* < 0.001). No gender differences were found in the Noticing, Emotion Awareness, and Body Listening scales.

**Table 6:** Gender differences in the Brief MAIA-2 scales.

Brief MAIA-2–2	Men (*n* = 143)		Women (*n* = 177)		*t*(318)	*p*	*d*
	*M*	*SD*	*M*	*SD*			
Noticing	9.49	3.42	9.79	3.48	− 0.78	0.438	− 0.09
Not Distracting	6.68	3.06	7.72	3.44	− 2.84	0.005	− 0.32
Not Worrying	7.64	3.24	6.56	3.64	2.76	0.006	0.31
Attention Regulation	8.95	3.15	7.41	3.67	3.97	< 0.001	0.45
Emotion Awareness	9.75	3.54	10.20	4.00	− 1.05	0.294	− 0.12
Self Regulation	8.41	3.21	6.31	3.92	5.17	< 0.001	0.58
Body Listening	8.27	3.50	8.19	3.62	0.22	0.830	0.02
Trusting	10.44	3.53	7.68	4.07	6.40	< 0.001	0.72
Total Interoception	69.63	14.42	63.86	15.99	3.35	< 0.001	0.12

The differences in the Brief MAIA-2 scales regarding sports participation were also examined (Table 7). Athletes scored significantly higher than Non-athletes in Noticing (medium effect size,* p* < 0.001), Attention Regulation (large effect size,* p* < 0.001), Emotion Awareness (medium effect size,* p* < 0.001), Self Regulation (large effect size,* p* < 0.001), Body Listening (medium effect size, *p* < 0.001), Trusting (large effect size,* p* < 0.001), and Total Interoception (large effect size,* p* < 0.001). In contrast, Non-athletes presented higher scores on the Not Distracting scale (medium effect size,* p* < 0.001). Athletes and Non-athletes did not differ in the Not Worrying scale of the Brief MAIA-2.

**Table 7:** Differences in the Brief MAIA-2 scales between Athletes and Non-athletes.

Brief MAIA-2–2	Non-athletes (*n* = 167)		Athletes (*n* = 156)		*t*(318)	*p*	*d*
	*M*	*SD*	*M*	*SD*			
Noticing	8.71	3.73	10.67	2.78	− 5.33	< 0.001	− 0.59
Not Distracting	8.18	3.67	6.28	2.53	5.39	< 0.001	0.60
Not Worrying	7.14	3.78	6.87	3.19	0.71	0.477	0.08
Attention Regulation	6.73	3.74	9.47	2.69	− 7.54	< 0.001	− 0.84
Emotion Awareness	9.00	4.24	11.14	2.90	− 5.26	< 0.001	− 0.59
Self Regulation	5.70	3.85	8.83	2.94	− 8.17	< 0.001	− 0.91
Body Listening	7.33	3.92	9.08	2.95	− 4.52	< 0.001	− 0.50
Trusting	7.12	4.29	10.74	2.87	− 8.86	< 0.001	− 0.99
Total Interoception	59.90	16.49	73.08	11.11	− 8.37	< 0.001	− 0.93

### Measurement invariance across genders and sports participation

A series of multigroup confirmatory factor analyses (MGCFA) to compare the latent means of the Brief MAIA-2 in the bi-factor (S·I – 1) model (assumed as the best in the study sample). Measurement invariance was examined across genders and sports participation (Table 8). The baseline model was established by running the CFA multiple times to assess configural, metric, scalar, and strict measurement invariance. These models demonstrated overall a good fit, considering χ^2^/(*df*), SRMR, RMSEA, and CFI. Although changes in CFI were significant, they were not supported by changes in RMSEA, which were very small^60^. Therefore, gender and sports participation invariance was confirmed in the study sample (*N* = 323).

**Table 8:** Measurement invariance across genders and sports participation: CFA models comparison (*N* = 323).

Models for gender	χ^2^/(*df*)	SRMR	RMSEA (90% CI)	CFI	Δχ^2^	ΔRMSEA	ΔCFI
0. Baseline	2.128	0.048	0.059 (0.051, 0.067)	0.946			
1. MI configural	1.804	0.046	0.050 (0.044, 0.056)	0.924			
2. MI metric	1.810	0.054	0.050 (0.045, 0.056)	0.916	0.006	0.000	–0.008
3. MI scalar	1.972	0.055	0.055 (0.050, 0.061)	0.893	0.162	0.005	–0.023
4. MI strict	2.005	0.070	0.056 (0.051, 0.159)	0.875	0.033	0.001	–0.018
0. Baseline	2.128	0.048	0.059 (0.051, 0.067)	0.946			
1. MI configural	1.722	0.058	0.047 (0.041, 0.053)	0.922			
2. MI metric	1.725	0.066	0.048 (0.042, 0.053)	0.914	0.003	0.008	0.008
3. MI scalar	1.922	0.067	0.054 (0.048, 0.059)	0.885	0.197	0.001	–0.007
4. MI strict	2.180	0.139	0.061 (0.056, 0.065)	0.834	0.258	0.072	0.071

## Discussion

The present study examined the factor structure and validation of the 24-item Brief MAIA-2 questionnaire. Many previous studies showed that previous versions of MAIA (i.e., the 32-item MAIA and 37-item MAIA-2) have problems with stabile factor structure and model fit^29,32,35,38,39,41,43,44,46–52^ and inappropriate reliability in some scales^29,31–33,35–38,41,43,45,48–50,52,53^, in particular Noticing, Not distracting, Not worrying, and Trusting. The appropriate CFA fit indices of the MAIA 8-factor structure were found only in Norwegian^42^ and Chinese^45^ studies (but with some correlations between residuals, as indicated by modification indices). Our research showed that the Brief MAIA-2 version is free of most previous issues. After excluding some items with poor factor loading, reliability, and shared variance, the questionnaire improved in all parametric properties. Reliability was appropriate for all scales (excluding Noticing). It ranged between 0.67 for Noticing and 0.89 for Trusting in Cronbach's α. Regarding McDonald's ω, reliability ranged between 0.67 for Noticing and 0.90 for Trusting. Since 0.67 or 0.68 is close to 0.70 as a threshold, this reliability value may be considered acceptable^57,64^. It's also important to consider the specific context of a measurement. In some research fields, lower reliability indices might be more acceptable due to the inherent difficulty in achieving high internal consistency among items. Various factors, including the number of items in a scale and the nature of the measured construct, can influence a low alpha. A low alpha might suggest that the construct is multidimensional or that certain items in the scale do not correlate well with others. The acceptability of a Cronbach's alpha of 0.60–0.70 depends on the context, the purpose of measurement, and other relevant factors^64^.

We fully confirmed using CFA that the 8-factor structure is appropriate for the data and fits the bi-factor (S·I – 1) model well. The results of the present study suggest that the general factor of interoception is appropriate as well as eight subscales (3-item each): Noticing (items 2, 3, and 4 of the original 37-item MAIA-2 version), Not Distracting (items 6, 9, and 10), Not Worrying (items 11, 12, and 15), Attention Regulation (items 17, 19, and 20), Emotional Awareness (items 25, 26, and 27), Self Regulation (items 29, 30, and 31), Body Listening (items 32, 33, and 34), and Trusting (items 35, 36, and 37). Moreover, for the first time (to the best of our knowledge), we performed confirmatory composite analysis to examine the convergent and discriminant validity of the Brief MAIA-2. We showed that all scales (except Noticing) have good reliability and convergent validity (considering CR and AVE) and that all scales demonstrate appropriate discriminant validity (regarding both intercorrelation structure and HTMT). Although all scales were weakly to moderately intercorrelated, the scale Not Worrying was only weakly associated with Noticing, Emotional Awareness, and Body Listening and unrelated to the other scales, including the total interoception score. Therefore, this scale can be more associated with another psychological construct (e.g., neuroticism, anxiety, depression) than interoception. More studies should be performed in the future to verify this hypothesis. On the other hand, total Interoceptive Awareness was the most highly related to Self Regulation, Trusting, Attention Regulation, and Body Listening, which confirms the assumptions of the multidimensional interoceptive awareness measurement.

The findings indicate that the Brief MAIA-2 bifactor structure is invariant across genders and sports participation. The present result is partially consistent with previous studies^40,51^, which showed gender invariance in the MAIA questionnaire. Furthermore, Fekih-Romdhane et al.^40^ demonstrated that men outperform women in mean scores of the Not Worrying and Attention Regulation scales of the MAIA-2, consistent with the present research. However, in our study, men scored also higher than women in Self Regulation, Trusting, and Total Interoception (besides the Not Worrying and Attention Regulation), and they scored lower in the Not Distracting scale compared to women. Only three scales did not differ between genders: Noticing, Emotion Awareness, and Body Listening. In contrast, Fiskum et al.^42^ found that women scored significantly lower than men in the Not Worrying while higher in the total MAIA-2 and Trusting scales. The inconsistency between our and the previous^42^ studies may result from the other version of the scale (37-item MAIA-2) or cultural differences. Therefore, more research is necessary to explain this issue.

The socialization process can explain gender differences in interoceptive and emotional abilities. According to the His-and-Hers Model of Perceptual Cue Use^5^, linguistic socialization contributes to gender differences by tending to label changes in internal state as physiological or emotional. Women and men use distinct interoceptive cues for bodily experiences. Based on internal cues, men identify physiological states and changes more accurately than women. In contrast, women use external situational cues to detect their internal states of the body (emotions and sensations) to a greater extent. A recent meta-analysis showed sex differences in interoceptive accuracy across cardiac, respiratory, and gastric domains^65^. In particular, men demonstrate better interoceptive accuracy in cardiac tests. However, this effect was not confirmed in gastric tasks, while respiratory tasks showed mixed effects. Instability or moderate heterogeneity was found in these two domains. Alfano et al.^4^ showed that women scored significantly higher than men in the Self-Awareness Questionnaire (SAQ). Also, significant correlations were found between self-reported interoceptive awareness and brain functional connectivity (FC) in the salience network and fronto-temporo-parietal brain areas. These findings support the idea that women show higher levels of attention to interoceptive sensations than men, which may be related to common inter-network areas that promote a sense of self-formation.

We also found differences in mean scores of the Brief MAIA-2 scales between people involved in sports activity and non-athletes. In particular, athletes outperform non-athletes in the Total Interoception score and all subscales, except the Not Worrying and Not Distracting. A large effect size was presented for these differences in Attention Regulation, Self Regulation, Trusting, and Total Interoception. However, it is important to note that non-athletes scored higher than athletes in Not Distracting (with medium effect size). The present result is consistent with previous studies. Almrcha et al.^9^ showed that people engaged in co-designed exercise intervention (they decided about the selected physical activities and the effort regulation) scored higher in almost all MAIA scales (except Not Worrying). Also, interoceptive awareness improved in three MAIA scales (Emotional Awareness, Self Regulation, and Body Listening) among war veterans with post-traumatic stress disorder (PTSD) symptoms after participating in a 12-week integrative exercise intervention program^12^. Wallman-Jones et al.^10^ indicate moderate and vigorous physical activity-based interventions can increase cardiac interoceptive accuracy among university students. Physical activity correlated positively with children's interoceptive awareness related to emotion and physical energy after Yoga intervention^11^. Elite world-class athletes (sprinters and distance runners) scored higher than non-athletes in Trust, Attention regulation, and Self-regulation of the MAIA^14^. Furthermore, professional dancers demonstrated a higher interoceptive accuracy in the heartbeat perception task than in the inactive control group^13^. Moreover, interoceptive accuracy increased with dance experience (measured in years).

Participation in physical activity and sports benefits interoceptive sensibility by increasing interoceptive awareness due to intensive training based on mindfulness, biofeedback, and other methods that increase the accuracy of interoceptive awareness. Wallman-Jones et al.^8^ explained that according to modern models of exercise regulation, top-down neural processes continuously monitor the body's physiological state to ensure that allostasis is maintained. The regulation of physical effort is based on interoceptive processes, including bottom-up interference affecting higher-order processes. Physical activity allows manipulation of afferent stimuli reaching the interoceptive system, which seems to optimize the integration of early sensory stimulation with later affective responses. Understanding how interoceptive processes are shaped by physical activity can explain the impact of interoceptive deficits on mental health and well-being^8^.

### Limitations of the study

Although the findings have many strengths, some weaknesses limit the generalization. First, the study design includes online questionnaires, limiting the representativeness of the general population. Since the validation was performed in a non-clinical population, it is unknown whether the same results would be achieved in clinical samples. Concurrent validation in this study is based on self-report gender and objective sport participation criteria. When measuring interoceptive awareness, it's important to consider the multidimensional nature of the concept, as it involves both accuracy in perceiving physiological sensations and subjective awareness of those sensations. Researchers can use a combination of objective and subjective measures to provide a more comprehensive understanding of an individual's interoceptive awareness^27,38^. Therefore, future studies could extend the present research, using various methods to assess interoception. Overall, more studies are necessary, including correlations with other interoceptive awareness measures, like self-report questionnaires, behavioral tests, and psychophysiological methods, in a more representative sample for the general population and various clinical groups. Also, the present study was performed in Poland, and the results may not be generalized to other countries and languages. More cross-cultural research is required to confirm the present validation of the Brief MAIA-2, considering the socialization process and environmental effects on interoception development in various countries and geographic regions.

## Conclusions

Brief MAIA-2 shows a good 8-factor structure with a higher-order overall interoceptive awareness index. We have determined that the 24-item MAIA-2 questionnaire is valid and reliable for measuring interoceptive sensitivity in a non-clinical sample. Although there are differences in the mean scores on Brief MAIA-2 scales by gender and participation in sport, the two-factor structure is invariant across gender and participation in sport. The abbreviated MAIA-2 scale can be widely used in scientific research, in particular, to examine changes in sensitivity and interoceptive awareness within specific therapies in clinical groups, as well as a result of mindfulness and physical activity training in a healthy population.

### Supplementary Information

Supplementary Information.

## Data Availability

All data generated or analyzed during this study are included in this published article [and its supplementary information file].
